# Safety Indices during Fetal Echocardiography at the Time of First-Trimester Scan Are Machine Dependent

**DOI:** 10.1371/journal.pone.0127570

**Published:** 2015-05-27

**Authors:** Dragos Nemescu, Anca Berescu, Mircea Onofriescu, Dan Bogdan Navolan, Cristian Rotariu

**Affiliations:** 1 Department of Obstetrics and Gynecology, University of Medicine and Pharmacy “Gr.T.Popa”, Iasi, Romania; 2 “Cuza Voda” Obstetrics and Gynecology Hospital, Iasi, Romania; 3 Department of Obstetrics and Gynecology, University of Medicine and Pharmacy ''Victor Babes'', Timisoara, Romania; 4 Department of Biomedical Sciences, University of Medicine and Pharmacy “Gr.T.Popa”, Iasi, Romania; University of Nebraska Medical Center, UNITED STATES

## Abstract

The aim of our study was to evaluate the thermal index (TI) and mechanical index (MI), during the assessment of the fetal heart at the time of first-trimester scan, with different ultrasound machines. This was part of an observational study conducted in patients undergoing routine first-trimester screening. Cases were examined with Voluson E8 or 730Pro scanners using 2–8 MHz transabdominal probes. TI and MI were retrieved from the saved displays while in gray mode, color flow mapping and pulsed-wave (PW) Doppler examinations of the fetal heart and also from the ductus venosus (DV) assessment. We evaluated 552 fetal cardiac examinations, 303 (55%) performed with Voluson E8 and 249 (45%) with Voluson 730Pro ultrasound machines. The gray-scale exam of the heart and the PW Doppler DV assessment had TI values significantly lower for the Voluson E8 group (median, 0.04 vs. 0.2 and 0.1 vs. 0.2, respectively). The MI values from gray-scale and color flow mapping of the heart were significantly lower (median, 0.6 vs, 1.2 and 0.7 vs. 1) and for PW Doppler exam of the tricuspid flow were significantly higher (median 0.4 vs. 0.2) in the Voluson E8 group. The TI values from Doppler examinations of the heart, either color flow or PW imaging and MI values from DV assessment were not significantly different between the two groups. A different (newer) generation of ultrasound equipment provides lower or at least the same safety indices for most of the first-trimester heart examinations.

## Introduction

There is an increased interest in evaluation of the fetal heart while in the first trimester of pregnancy. Even if a detailed examination is impossible, a basic assessment of the fetal heart at 11 to 14 weeks of gestation has been investigated by some groups. [1, 2] Now, there are several series reporting the detection of major cardiac defects.[3, 4] At this gestational age, color flow mapping has a dominant role, improving the visualization of chambers and vessels as well as demonstrating flow direction. There are often associated the tricuspid flow and ductus venosus (DV) pulsed-wave (PW) Doppler evaluation as a part of aneuploidy screening [5], improving the early detection of heart defects.[6]

As a form of energy, the ultrasounds have the potential for causing bioeffects by heating and cavitation. Ultrasound machines have to display the thermal index (TI) and mechanical index (MI) on the screen during examination, as an indication of the likelihood of ultrasound-induced bioeffects. They encourage the end-user to become aware of safety issues and enable the application of ALARA (as low as reasonably achievable) principle. The TI expresses the potential for a rise in temperature along of the ultrasound beam. The MI indicates the potential for the ultrasound to induce cavitation. However, because of the absence of a gas-liquid interface in the utero, this effect has not been documented in mammalian fetuses and there is no direct evidence to date as to whether or not this effect can occur in humans.[7]

The levels of TI and MI are generally low while in nuchal translucency measurement [7] and fetal echocardiography at 11–13 weeks’ gestation.[8] According to the current recommendation, TI should not exceed a value of 1.0 for the first-trimester Doppler examinations.[9]

However, evidence on the safety of the ultrasound is insufficient, and it is considered that the fetus it is at risk of exposure to high ultrasound levels during its early development, when it is sensitive to external influences. Therefore, caution has been recommended.[10]

Our objective was to analyze the levels of TI and MI during fetal echocardiography at the time of the first-trimester scan, for different ultrasound systems.

## Material and Methods

This was part of a prospective observational study in women attending for first-trimester ultrasound screening at our care center, between December 2009 and May 2013. We retrieved from the saved displays TI (for soft tissues) and MI values from the gray mode, color flow mapping and pulsed-wave (PW) Doppler ultrasound examinations of the heart and from the assessment of the ductus venosus. Written informed consents were obtained from participants. The study protocol was approved by the local Ethics Committee (“Cuza Voda” Obstetrics & Gynecology Hospital). Patients were enrolled in a consecutive manner. Viable singleton pregnancies with a crown-rump length (CRL) of 45 to 84 mm were included.

All examinations were performed transabdominally, using Voluson E8 (RAB4-8D probe) or Voluson 730 (RAB4-8L probe) ultrasound systems (GE Healthcare, Zipf, Austria) by a single trained operator, certified by the Fetal Medicine Foundation, with more than five years of experience in obstetrical scanning and performing 1000 fetal anomaly scans per year.

The image of the fetal thorax was magnified using the machine zoom (HD zoom) so that it occupied most of the image. Usually, after examination using B-mode, the operator interrogated the tricuspid flow and then applied the color flow mapping on the same zoomed heart window. Systematically, we evaluated the 4CV—ventricular filling, right and left ventricular outflow tracts, crossover of the great arteries, 3-vessel and trachea view using color flow mapping as previously described.[2, 11] The cine-loop facility of the ultrasound machine was used to identify studied fetal heart segments. If the image quality was poor, the operator could choose to switch to a transvaginal approach, and these cases were not included within the study. The tricuspid and ductus venosus flows were evaluated as previously described.[12, 13]

We started with specific presets defined by the manufacturer for first-trimester evaluation of the heart, and these were then adjusted and saved as the study progressed. For the ductus venosus, we used distinct settings. Depth, focus, overall gain (post-processing) and power were adjusted as necessary. The sonographer was unaware of the data followed by the study and fully complied with the guidelines on the safe use of ultrasound at this gestational age.[14] When the operator was satisfied by the image, this was saved. Thus, the images of structures defined by the operator as visualized were stored in the ultrasound equipment and subsequently exported.

When reviewing the data, we found that Voluson E8 machine does not show, by design,[15] the thermal indices (TIs) from gray-scale examinations of the heart with values below 0.04 and display them as 0.0. We assumed that the energy emission cannot be null. Therefore, for statistical purposes and because we were looking for maximum values of indexes, these were re-coded at 0.04.

Statistical analyses were performed using SPSS program, version 21.0 (SPSS, Chicago, IL). Continuous variables were compared between the two ultrasound machine groups using the Student’s and Mann–Whitney U tests. The acoustic output differences inside of the same machine group were assessed using the Wilcoxon signed-rank test for two related samples. The outliers were excluded from statistical tests. The level of significance was set at p <0.05.

## Results

A total of 552 fetal cardiac examinations were included. 303 (55%) cases were assessed with the Voluson E8 machine and 249 (45%) with the Voluson 730 Pro. Characteristics of the two groups are summarized in Table 1. Women evaluated with Voluson E8 were significantly older and had a shorter transducer-to-heart distance. We excluded from the analysis 7 fetuses with cardiac malformations, 11 cases with aneuploidies and 5 cases with major fetal anomalies.

**Table 1:** Baseline characteristics of patients according to the type of ultrasound machine used for evaluation of the fetal heart.

	Voluson E8 (n = 303)	Voluson 730 Pro (n = 249)	p
**Maternal age, years**	30.4 (4.5)	29.0 (4.5)	<0.001
**CRL, mm**	62.8 (9)	62.7 (9.5)	NS
**GA at US, weeks**	12.4 (0.7)	12.5 (0.7)	NS
**BMI, kg/m^2^**	22.9 (3.7)	23.1 (3.8)	NS
**Transducer-heart distance, cm**	6.3 (1.2)	6.6 (1.3)	0.004

Data given as mean (standard deviation) or n (%). All comparisons were made using t-Student test. BMI, body mass index; GA, gestational age; US, ultrasound.

The maximum values for mechanical and soft-tissue thermal indices sampled during gray-scale and Doppler evaluation of the fetal heart and for the ductus venosus assessments are presented in Table 2. We found significant differences between the two ultrasound machine groups, and we considered them separately.

**Table 2:** The maximum acoustic output during evaluation of the fetal heart at the time of the first trimester scan.

	B-Mode	Color Doppler	PD TR	PD DV
**Thermal Index**					
**Voluson E8**	0.04 (0.04–0.2)*	0.2 (0.2–0.2)	0.4 (0.2–0.4)	0.1 (0.1–0.3)
**Voluson 730**	0.2 (0.1–0.2)	0.2 (0.2–0.3)	0.4 (0.2–0.5)	0.2 (0.1–0.5)
**p**	<0.001	NS	NS	<0.001
**Mechanical Index**					
**Voluson E8**	0.6 (0.5–1.1)	0.7 (0.5–1.1)	0.4 (0.2–0.4)	0.4 (0.4–0.4)
**Voluson 730**	1.2 (0.8–1.2)	1.0 (0.6–1.2)	0.2 (0.2–0.8)	0.4 (0.3–0.5)
**p**	<0.001	<0.001	<0.001	NS

* recoded data.

Data are given as median (minimum—maximum). All comparisons were made using Mann–Whitney U test. PD TR, pulsed-wave Doppler tricuspid flow assessment; PD DV, pulsed-wave Doppler from the ductus venosus assessment.

Comparison of the TI showed that this was significantly lower in the Voluson E8 group, for the heart examinations performed in gray-scale (Table 2). TIs values from Doppler exams, either color flow or PW imaging, were not significantly different between the two ultrasound machines. The TIs from color flow mapping were constant within the Voluson E8 group and for the majority (97.8%) of the Voluson 730 group, the rest being greater values. In the case of PW Doppler assessment of the ductus venosus, TIs were significantly lower for Voluson E8 group. Here, the majority (98.3%) of the values were also constant.

Mechanical indices (MIs) from gray-scale and color flow mapping of the heart were significantly lower, but from PW exam of the tricuspid flow were significantly higher among the Voluson E8 group (Table 2). The MI values from the DV assessment were not significantly different between the two ultrasound machines.

There are significant differences in the safety indices between examinations of the fetal heart, inside of the same machine group (related samples Wilcoxon signed-rank test, p<0.01). For Voluson E8, there was a significant increase of TI from B-mode studies to color flow mapping and then in PW assessment of the tricuspid flow (Table 2). This phenomenon did not appear in the case of Voluson 730 machine, where B-mode and color Doppler examination had the same TI. For both machines, the TI values from the DV pulsed Doppler assessment were significantly lower than those from the tricuspid flow evaluation. The ductus venosus TIs were comparable (but higher) with those from the color flow mapping of the heart for the Voluson 730 group, but significantly lower than them, for the Voluson E8 group.

In the Voluson 730 group, the MI showed a significant, continuous, decrease from the B-mode to the color flow Doppler and then to the pulsed wave Doppler studies (Table 2). Moreover, the TIs from the power Doppler assessment of the ductus venosus were significantly higher than those from the tricuspid flow evaluation. The evolution was different for the Voluson E8 group, where the MI values of the B-mode were lower, but comparable, than those from the color flow mapping and both values were higher than those from the pulsed wave Doppler studies. The MI values of the power Doppler assessment of the tricuspid flow and ductus venosus were not different in this group.

The soft-tissue thermal and mechanical indices were remarkably stable during the evaluation of the same fetus, with a maximum variation of 0.1 for all four types of exams.

## Discussion

This study demonstrated that newer generation of ultrasound equipment (Voluson E8 vs 730) provides lower thermal indices for gray-scale exam of the heart, ductus venosus interrogation and lower mechanical indices for gray-scale, color mapped imaging of the heart and evaluation of the tricuspid flow. For some specific examinations of the heart, safety indices had a low variability, some of them being even constant.

There were significant variations of the safety indices inside of the same machine group. For the E8 system, the TI increased from B-mode studies to color flow mapping and then in PW assessment of tricuspid flow. In the 730 group, this increase was present only for tricuspid flow assessment, but not for B-mode and color Doppler, which had the same TI values. The TI from PW Doppler assessment of the ductus venosus was lower than those from the evaluation of the tricuspid flow.

For E8 ultrasound system, we reported recently very similar values for safety indices, on first-trimester fetal echocardiography.[8] These were acquired on a larger, but with the same selection criteria, no-anomaly group. Actual data proves the robustness of our methodology.

By definition, soft-tissue thermal index depends on the current acoustic output power emitted from the transducer, which has been shown by previous measurements[16] to increase from B-mode, to color flow mapping and then in pulsed-wave Doppler studies. Besides of the overall power intensity control, ultrasound exposure is influenced by scanning parameters. The window for heart examination is small (2/2 cm), with dimensions appropriate to the fetal thorax and therefore, relatively unchanged at 11–13 gestational weeks. In our scanning conditions, we found that TI is very steady and does not change with probe-to-heart distance variation, even greater than 3 cm. In addition, the velocity scale related to pulse repetition frequency is quite stable, at 30–40 cm/s. The position and number of the focus induces a small variation, of maximum 0.1, in the TI values. For PW Doppler, the sample volume (gate) size is fixed to 3 mm and 0.7–1 mm, respectively for tricuspid flow and DV evaluation.[12, 13] Assuming that the calculation algorithms for safety indices are the same between ultrasound machines, these factors together explain the variation of the TI values between exams inside the same ultrasound machine group, their very little variability and may indicate that the acoustic power is lower for E8 machine. The improvement of the ultrasound equipment (advanced signal processing modules and better resolution) could also contribute to these results.

The equation for MI predicts that inertial cavitation is more likely at higher values of the peak rarefaction pressure in the beam and at lower frequencies. Previous studies in water showed that the peak rarefaction pressure is increasing from PW Doppler, B-mode and color Doppler exams, respectively.[16] In our scanning conditions, we found that MI correlates to windows size, pulse repetition frequency and is inversely related with depth (probe-to-heart distance) and focus position. Thus, scanning at higher frequencies could explain the lower MI for gray-scale and color Doppler examinations of the heart, resulted with Voluson E8.

We found a large proportion of safety indices with constant values (Table 2). This low variability was also described by others during fetal nuchal translucency evaluation.[7] For a better understanding of this phenomenon, we need two-digit displayed values. Moreover, this characteristic highlights the importance of adherence to the assessment protocol and suggests the potential for supplementary active reduction of these indices. Changes in the settings, that would allow a reduction of the safety indices, would be more easily implemented on a large scale.

We achieved spontaneously, without an intentional reduction in the power to reach a predefined level,[17] low values of thermal index, that never exceeded 0.5, during the routine fetal echocardiography at the time of first-trimester screening.

A limitation of our study was the fact that the depth (probe-to-heart distance) is significantly different, higher for Voluson 730 compared to E8. This difference is small (under 0.5 cm) and as was discussed earlier, in our scanning conditions, practically, it is unlikely it could induce a significant difference between safety indices. Thus, TI is very stable and does not change over a variation of the depth for more than 3 cm. MI is inversely related to probe-to-heart distance and should decrease because of an increased depth. A better, more precise differentiation could be achieved examining each patient with both scanners. The variation of the safety indices values arises from differences which are not considered separately here. However, it is difficult to guarantee depth, focus, windows size, frequency and other parameters of the ultrasound examination in human.

Our objective was to evaluate the values of TI and MI during the routine examinations of the heart and ductus venosus with the clear intent of not developing sophisticated calculations and equations, but offering clinical results. We found significant differences between the two ultrasound machine groups, and we had considered them separately. However, it is not sure that the results can be generalized to all scanners.

The MI is valid under conditions for the onset of inertial cavitation: the presence of bubble nuclei in the tissue. Therefore, it is highly improbable that cavitation can be generated at diagnostic levels within fetal soft tissues or fluids, in the absence of a gas-liquid interface in the tissues such as the lung or intestine, or without presence of gas-based ultrasound contrast agents.

There is no way to measure actual in situ exposure in human fetuses. TI is not directly correlated with actual temperature.[18] The TI can underestimate actual temperature and in a worst-case, temperature rise may be three times higher than the displayed value.[16] Therefore, even our study showed low values for indexes of the acoustic energy during fetal echocardiography at the time of the first-trimester scan; these results should be considered with caution because we don’t know for sure if they are low enough to be safe for the fetus.[10] Users should regularly check both indices while scanning and should adjust the machine controls to keep them as low as reasonably achievable (ALARA principle) without compromising the diagnostic value of the examination. Where low values cannot be achieved, examination times should be kept as short as possible.[19]

In conclusion, a different (newer) generation of ultrasound equipment provides lower or at least the same safety indices for most of the first-trimester heart examinations. For some specific exams of the heart, safety indices had a low variability, some of them being even constant.
